# Modified Hevein-like Peptide from *Amaranthus caudatus* as a Promising Agent Against Pathogenic *Candida* Species

**DOI:** 10.3390/pharmaceutics17111406

**Published:** 2025-10-30

**Authors:** Ekaterina I. Finkina, Anastasia A. Gerasimova, Olga V. Shevchenko, Ivan V. Bogdanov, Andrey A. Tagaev, Alexander D. Voropaev, Tatiana V. Ovchinnikova

**Affiliations:** 1 M.M. Shemyakin and Yu.A. Ovchinnikov Institute of Bioorganic Chemistry, Russian Academy of Sciences, 117997 Moscow, Russiaovch@ibch.ru (T.V.O.); 2 Moscow Center for Advanced Studies, 123592 Moscow, Russia; 3 G.N. Gabrichevsky Research Institute for Epidemiology and Microbiology, Admiral Makarov St. 10, 125212 Moscow, Russia

**Keywords:** fungal infections, *Candida albicans*, non-albicans *Candida* species, plant hevein-like peptides, mAc-AMP2, resistance, clinical isolates, antifungal activity, fungal cell adhesion, fungal biofilms

## Abstract

**Background/Objectives:** Currently, infections caused by fungi of the *Candida* genus remain a significant global health concern. The rising incidence of mycoses, coupled with the rapid emergence of fungal resistance, highlights the urgent need to search for new antifungal agents. Here, we obtained the recombinant hevein-like peptide from *Amaranthus caudatus* with two amino acid substitutions (F18W in the chitin-binding motif and M13A preventing the peptide from cleavage with cyanogen bromide during its biotechnological production). **Methods**: Antifungal potential of the modified hevein-like peptide, designated as mAc-AMP2, against susceptible and resistant strains of *Candida albicans* and non-albicans *Candida* species was studied. **Results:** We showed that mAc-AMP2 possessed anticandidal activities against all strains tested at nanomolar peptide concentrations. The presence of salts or serum affected the action of the peptide but its antifungal activity remained quite high. mAc-AMP2 exhibited anti-adherent properties and inhibited the formation of fungal biofilms. Using RP-HPLC, we demonstrated that degradation of the peptide in the presence of serum occurred rather slowly. mAc-AMP2 did not exhibit hemolytic and cytotoxic activities against the Caco-2 cell monolayer and peripheral blood mononuclear cells. Using flow cytometry, we demonstrated that the peptide at its high concentrations increased fungal membrane permeability. In resistance induction experiments, sensitivity of *C. albicans* toward mAc-AMP2 decreased over time, but restored after the peptide elimination. **Conclusions:** Taking into account all the data obtained, we suggest that the modified hevein-like peptide is a promising candidate for development of novel therapeutic agents to combat fungal infections caused by *C. albicans* and other *Candida* species.

## 1. Introduction

In recent years, the World Health Organization (WHO) has reported a rise in severe and chronic fungal infections, especially in immunocompromised individuals, along with an increasing occurrence of drug-resistant fungal strains [1]. Representatives of the *Candida* genus are the most common cause of fungal infections ranging from mild external to life-threatening systemic ones. *C. albicans* and *C. auris* are classified by WHO as fungal pathogens of a critical priority group [1]. But other non-albicans *Candida* species (NAC), such as *C. tropicalis*, *C. parapsilosis*, *C. krusei* (reclassified as *Pichia kudriavzevii*), and *C. glabrata* (reclassified as *Nakaseomyces glabrata*) have also emerged as significant threats in recent years [2]. At the same time, the list of conventional antimycotics is quite limited, and most of them exhibit serious side effects, frequent drug–drug interactions, and poor solubility [3]. Despite this, WHO data indicate that only four new antifungal drugs have been approved over the last ten years [4]. The combination of these factors necessitates the search for potential prototypes of new antifungal drugs.

Plant antimicrobial peptides (AMPs), which specifically target fungi and exhibit a low toxicity, are promising candidates for the development of new antimycotics. Among them, hevein-like peptides have been shown to effectively inhibit the growth of various phytopathogenic fungi [5,6]. Chitin, a fungal-specific component of the cell wall, is known to be the target of their action. These AMPs are small, cysteine-rich peptides containing a conserved chitin-binding motif (SXFGY, where X can be represented by any amino acid residue), which is also found in sequences of plant chitinases of various classes [5]. A potential application of hevein-like peptides in agriculture is actively being studied [7,8]; however, their effects on fungi pathogenic to humans remain largely unexplored.

It has been shown that hevein-like peptide Ac-AMP2, from the seeds of *Amaranthus caudatus*, possessed the pronounced activity against various phytopathogenic fungi [9]. Peptide consists of 30 amino acid residues including six cysteines. The spatial structure of Ac-AMP2 is represented by a short C-terminal α-helix and antiparallel β-sheets stabilized by three disulfide bonds [10]. It has been suggested that the antifungal action of the peptide is realized due to its chitin-binding capacity and chitinase activity [5]. Previously, the effect of various amino acid substitutions on the peptide affinity for chitin has been studied. It has been shown that the replacement of the 18th amino acid residue Phe for Trp led to an increase in the chitin-binding capacity of Ac-AMP2 [11,12,13]. Here, we studied the modified Ac-AMP2 (mAc-AMP2; VGECVRGRCPSGACCSQWGYCGKGPKYCGR, Mw 3162.67, pI 8.92) with the F18W substitution in the chitin-binding motif of the peptide along with the additional M13A substitution which protected the desired peptide during its production via cleavage of the fusion protein with cyanogen bromide.

The main goal of this study was to investigate the antifungal potential of mAc-AMP2 against susceptible and resistant strains of fungi of the *Candida* genus. For that purpose, collection strains as well as clinical isolates of *C. albicans*, *C. krusei*, *C. glabrata*, and *C. tropicalis* were used. We investigated the antifungal action of mAc-AMP2 and effects of various salts and serums on its activity. The antibiofilm activity of mAc-AMP2 as well as its capacity to prevent fungal adhesion to the epithelial monolayer and plastic surface were also demonstrated. The stability of the peptide to boiling and the presence of reducing agent and serum components were investigated. The ability of mAc-AMP2 to disrupt the fungal cell membrane was studied by using flow cytometry. Finally, resistance induction experiments were also performed using not only mAc-AMP2 but also other AMPs and caspofungin for comparison.

## 2. Materials and Methods

### 2.1. Materials

Here, we used different species of the *Candida* genus: collection strains of *C. albicans*—susceptible ATCC 18804 and resistant to azoles and anidulafungin ATCC 10231; resistant to azoles and anidulafungin clinical isolates of *C. albicans* 9.1 and 8.2; resistant to azoles and polyenes clinical isolates of *C. tropicalis* v13a4/2 and *C. glabrata* 252/2; resistant to azoles clinical isolate *C. krusei* 225/2 (Supplementary Materials, Table S1). All clinical isolates were obtained from patients with human immunodeficiency virus (HIV) infection and candidiasis and provided by the G.N. Gabrichevsky Research Institute for Epidemiology and Microbiology (Moscow, Russia).

Caspofungin (Sigma, St. Louis, MO, USA), voriconazole (Sigma, USA), and ebselen (Sigma, St. Louis, MO, USA), which exhibit antifungal activity, were used in checkerboard antifungal assay. Synthetic melittin (>98% pure) obtained in M.M. Shemyakin and Yu.A. Ovchinnikov Institute of Bioorganic Chemistry of the Russian Academy of Sciences (Moscow, Russia) was used for comparison in cytotoxicity assay.

Colorectal adenocarcinoma Caco-2 cells in a monolayer (ATCC HTB-37) and peripheral blood mononuclear cells (PBMCs, ATCC PCS-800-011) were used to evaluate cytotoxic effects of mAc-AMP2. The Caco-2 epithelial monolayer as an in vitro model of the intestinal barrier was also used for investigation of *C. albicans* cell adhesion.

Recombinant antimicrobial peptides (tobacco defensin NaD1 (UNIPROT Q8GTM0), human cathelicidin LL-37 (UNIPROT P49913), and β-defensin 2 (HBD2, UNIPROT O15263)) were obtained as described in [14].

### 2.2. Recombinant Production of Antimicrobial Peptide mAc-AMP2

The recombinant mAc-AMP2 was obtained by heterologous expression in *E. coli* cells mainly as described by us earlier for tobacco defensin NaD1 [14] (Supplementary Materials). The high quality of the recombinant peptide preparation was confirmed by SDS-PAGE, MALDI-TOF mass spectrometry, and CD spectroscopy (Supplementary Materials, Figures S3–S5, Table S3).

### 2.3. Antifungal Activity Assay

Microdilution method in 96-well flat-bottom microplates was used to study the antifungal activity of mAc-AMP2 mainly as described in [15]. Briefly, fungal cells at a concentration of 4 × 10^4^ cells/mL in the Sabouraud broth were mixed with equal volumes of serial dilutions of the peptide in water (final volume 100 μL and pH 6.0) and incubated at 30 °C for 24 h. Inhibition of fungal growth was assessed relative to the peptide-free control, based on absorbance at 630 nm and microscopic analysis. MIC_50_ and MIC were defined as minimal concentrations of the peptide, causing 50 and 100% inhibition of fungal growth. MFC was defined as minimum fungicidal concentration of mAc-AMP2 that produced no visible growth after plating the content of the wells with concentrations equal to or greater than the MIC onto Sabouraud agar supplemented with 2% glucose and 20 μg/mL chloramphenicol (Sigma, USA). The influence of various salts and serums on the activity of mAc-AMP2 towards *C. albicans* ATCC 18804 was investigated in the same way by using Sabouraud broth with 150 mM NaCl or 1.25 mM MgCl_2_ or 1.25 mM CaCl_2_ or 10% FBS or RPMI-1640 with 2% glucose, pH 7.2. To assess the effects of pH on mAc-AMP2 activity, peptide dilutions in 0.02% TFA or 50 mM NH_4_HCO_3_, pH 8.0, were added to the cell culture in Sabouraud broth (final pH 5.0 or 7.0, respectively). The effects of heating and reduction in disulfide bonds on the activity of the peptide towards *C. albicans* ATCC 18804 was also tested. For this purpose, mAc-AMP2 was heated at 99 °C for 15 min or incubated in the presence of 10 mM DTT for 30 min before antifungal assay. Tobacco defensin NaD1, whose structure is also stabilized by disulfide bonds, was used for comparison in these experiments. The effect of reduced L-glutathione (AppliChem, Darmstadt, Germany), a component of RPMI-1640 medium, at a final concentration of 3.25 μM, was also studied.

Each experiment was conducted twice in triplicate. The microplate wells were pre-treated with 0.1% BSA to minimize adsorption of antifungal agents.

### 2.4. Checkerboard Antifungal Assay

The combined action of mAc-AMP2 with other antifungal agents was studied similarly using the following strains of *C. albicans*: ATCC 18804, 8.2 or 9.1. Serial dilutions of the mixture of two combinable substances were added to the equal volumes of fungal cell suspensions in this case. The combined effects of antifungal substances were assessed using the fractional inhibitory concentration index (FICI) which was calculated as ([A])/([MIC_A_]) + ([B])/([MIC_B_]); where [MIC_A_] and [MIC_B_] indicate the MICs of mAc-AMP2 and the other antifungal compound when used alone, and [A] and [B] represent their concentrations in combinations yielding equivalent inhibitory effects. Caspofungin or ebselen in 1% DMSO as well as voriconazole in 1% methanol were tested in these experiments.

### 2.5. Antibiofilm Activity

The effects of mAc-AMP2 on biofilm formation by *C. albicans* ATCC 10231, 8.2 or 9.1 cells in Sabouraud broth were investigated mainly as described [14]. Yeast cells were used at final concentration of 10^6^ cells/mL and formed biofilms in the presence or without peptide at 37 °C for 24 h. Formed biofilms were washed twice with PBS to remove non-adhered cells and cell viability was determined after microplates incubated for 6 h by using the resazurin-test. The fluorescence of resorufin was recorded using a 535/595 filter. Each experiment was conducted twice in triplicate. BIC_50_ and BIC represent the minimal peptide concentrations required to inhibit biofilm formation by 50% or 100%.

The effects of mAc-AMP2 on biofilms formed by *C. albicans* strains ATCC 10231, 9.1 or 8.2 were studied in a similar way [14]. However, in this case, serial dilutions of the peptide in Sabouraud broth were added to already formed biofilms and incubated at 37 °C for 24 h.

### 2.6. Hemolytic Assay

The hemolytic activity of mAc-AMP2 was studied in 96-well microplates by using fresh human red blood cells (hRBC) in PBS at a concentration of 4% (*v*/*v*). Experiments were performed with the hRBC from blood samples collected from healthy donors by certified medical personnel upon informed written consent. All procedures were approved by the Ethics Committee of the Institute of Experimental Medicine (protocol 1/20 of 2/27/2020) and comply with the ethical principles of the Declaration of Helsinki. Serial two-fold dilutions of mAc-AMP2 in PBS at final concentrations from 0.19 to 25 μM were added to the cells. After incubation for 2 h at 37 °C hemoglobin release was assessed by measuring the optical density of supernatants from plate wells at 405 nm. Cells without peptide or in the presence of 0.1% non-ionogenic detergent Triton X-100 were used as negative (c^−^) and positive (c^+^) controls, respectively. The percentage of hemolysis was calculated as ((ODs − ODc^−^)/(ODc^+^ − ODc^−^)) × 100%. The experiment was carried out twice in triplicate. Melittin, membrane-active peptide from honeybee venom, was also used in these experiments.

### 2.7. Cytotoxicity Assay

The cytotoxic effects of mAc-AMP2 towards human peripheral blood mononuclear cells (PBMCs) or Caco-2 cells in a monolayer were tested in 96-well plates by using resazurin assay [16]. Melittin which exhibits non-specific cytotoxicity was also used in these experiments for comparison. For that purpose, Caco-2 cells in a monolayer in DMEM/F12 (1:1) medium or 2 × 10^6^ PBMCs per well in RPMI-1640 were incubated with serial dilutions of mAc-AMP2 in total volume of 100 μL for 24 h in a CO_2_ incubator (5% CO_2_, 37 °C). Then 10 µL of resazurin at a concentration of 0.7 mM was added and the incubation was continued for 4 h. Untreated cells and cells in the presence of 0.1% Triton X-100 were used as negative and positive controls, respectively. The cell viability was estimated by resorufin fluorescence registered at 595 nm as (F_sample_/F_control_) × 100%. Wells containing no cells were used to subtract background fluorescence. This experiment was carried out twice in duplicate.

### 2.8. Membrane Permeability Assay

Membrane permeability assay was performed by flow cytometry using a Novocyte 2060R flow cytometer (ACEA Biosciences Inc., San Diego, CA, USA). For that purpose, intercalating dye propidium iodide (PI, Biotium, Fremont, CA, USA) uptake was registered using a 488 nm blue laser. *C. albicans* ATCC 18804 cells at a final concentration of 2 × 10^4^ cells/mL in Sabouraud broth were incubated at 30 °C for 2 or 20 h at a volume of 4 mL in the presence of or without mAc-AMP2 at concentrations of 0.25 × MIC, MIC and 4 × MIC. Tobacco defensin NaD1 and its combination with mAc-AMP2 at the concentration of 0.25 × MIC (for both antifungal agents) were used in these experiments. After incubation, the cells were centrifuged at 1600× *g* for 10 min, resuspended in 300 μL PBS, and stained with PI at the concentration of 4 μg/mL for 20 min. For each sample, at least 15,000 events were recorded. The obtained data were processed using NovoExpress Software v.1.2.4 (ACEA Biosciences Inc., San Diego, CA, USA). Untreated and heat-killed at 99 °C for 15 min cells of *C. albicans* were used as referential live and dead fungal cells.

### 2.9. Stability in Serum

The stability of mAc-AMP2 in serum was investigated mainly as described in [17]. For that, 16.25 μL of mAc-AMP2 at a concentration of 4 mg/mL was mixed with 244 μL of 25% fresh human serum in PBS, pH 7.4, and incubated for 0, 4, and 24 h at 37 °C. Urea solution was added to the final concentration of 3.75 M. Then, serum proteins were precipitated with 7% trifluoroacetic acid (TFA) for 30 min at 4 °C and centrifuged at 13,000 rpm for 20 min. RP-HPLC in a linear gradient of acetonitrile from 5 to 80% for 45 min in the presence of 0.1% TFA (Reprosil-pur C18-AQ, Dr. Maisch GmbH, Ammerbuch, Germany) were performed to analyze peptide content in supernatants. MALDI-TOF mass spectrometry was used to confirm the presence of mAc-AMP2. For negative control, a similar method was used for mAc-AMP2 in PBS without serum (Supplementary Materials, Figure S6). This experiment was carried out twice.

### 2.10. Prevention of Adhesion to the Epithelial Monolayer

The influence of mAc-AMP2 on the adhesion of *C. albicans* ATCC 10231, 8.2 or 9.1 cells to the Caco-2 cell monolayer as an in vitro model of the intestinal barrier mainly was studied as described [14]. The Caco-2 cell monolayer in 96-well flat-bottom plates in RPMI-1640 with 0.2% glucose and sodium bicarbonate was prepared before the test. *C. albicans* cells with or without peptide in the same medium were added to the wells at a final volume of 100 µL (5 × 10^4^ cells/well). After incubation for 1.5 h at 37 °C in a CO_2_ incubator, unattached *C. albicans* cells were removed by washing the wells twice with PBS. Adherent cells were detached with trypsin/EDTA solution (PanEco, Moscow, Russia), diluted 100-fold with PBS and plated on Sabouraud agar with glucose and chloramphenicol. A total of 10% FBS was used to inhibit trypsin activity. The plates were incubated at 37 °C for 24 h, after that the grown fungal colonies were counted. Cell adhesion was quantified as ((N × 100)/5 × 10^4^) × 100%, where N represents the average number of adhered cells on the plate. Inhibition of cell adhesion was calculated as ((N − N_control_)/ N) × 100%, where N_control_ represents the average number of adhered cells in control without peptide. This study was performed twice in triplicate.

### 2.11. Prevention of Adhesion to the Plastic Surface

*C. albicans* 8.2 cells with or without antifungal agent (mAc-AMP2, NaD1 or caspofungin) in Sabouraud broth were added to the wells at final volume of 100 µL (5 × 10^5^ cells/well). The plates were incubated for 1.5 h at 37 °C; after that, plate wells were washed twice with PBS to remove unattached *C. albicans* cells. Adherent cells were detached by microplate incubation for 45 min with trypsin/EDTA solution. Enzymatic activity was stopped by an addition of 10% FBS. After that the well contents were scraped, diluted 100-fold with PBS, and plated on Sabouraud agar with glucose and chloramphenicol. In this case, adhesion was calculated as ((N × 100)/5 × 10^5^) × 100%, and adhesion inhibition was calculated as described above. These experiments were carried out twice in triplicate.

### 2.12. Resistance Induction Experiments

The resistance was induced by the repetitive treatment of fungal cells with mAc-AMP2 at sub-MIC concentrations. For comparison, the echinocandin caspofungin, human cathelicidin LL-37, and tobacco defensin NaD1 were used in these experiments. *C. albicans* ATCC 18804 or ATCC 10231 at a concentration of 4 × 10^4^ cells/mL were added to serial dilutions of antifungal agents. The 96-well microplates were incubated overnight at 30 °C and MIC values were determined as described above. Further, the contents of the wells containing antifungal agents at the concentration of 0.25 × MIC were diluted to a concentration of 4 × 10^4^ cells/mL with fresh Sabouraud broth and used as a starting culture for the next round of selection. Sequential passaging followed by MIC determination continued for 24 days. Fungal cells of both strains capable of growing at the highest concentration of mAc-AMP2 at the end of experiment were passaged thrice on agar plates in the absence of the peptide. After that whole cultures as well as single colonies were used for MICs determination to confirm the stability of acquired resistance to the peptide.

### 2.13. Calcofluor White Binding Assay

Chitin content determination in *C. albicans* ATCC 18804 cells untreated and treated by mAc-AMP2 was performed using a fluorescent stain Calcofluor White (CFW, Thermo Scientific, Lenexa, KS, USA) that binds to chitin in the cell walls of fungi [18,19]. Polyene antimycotic amphotericin B (AmB) was also used in these experiments. *C. albicans* cells at a concentration of 4 × 10^5^ cells/mL were grown in Sabouraud broth in the absence or presence of mAc-AMP2 at the final concertations of 3.13, 6.25, and 12.5 μM with shaking at 30 °C for 4 h. Cell cultures were harvested, washed twice with PBS, and stained with 20 μg/mL CFW in PBS for 30 min. After that stained cells were washed three times with PBS and the cell density of each group was adjusted to 10^6^ cells/mL using Cell Counter LUNA-II (Logos Biosystems, Anyang-si, Republic of Korea) and trypan blue. Unstained or stained untreated by antifungal agent cells were used as negative and positive controls, respectively. Fluorescence intensities were measured at 20 °C using a Hitachi F-2710 spectrofluorometer (Hitachi High Technologies America Inc., Pleasanton, CA, USA) with excitation and emission wavelengths of 350 nm (10 nm width) and 435 nm, respectively. Results were expressed as % of fluorescence intensity in comparison to positive control. This study was performed twice in triplicate.

## 3. Results and Discussion

### 3.1. mAc-AMP2 Is Effective Against Susceptible and Resistant Strains of Fungi of the Candida Genus

Here, we performed the study of antifungal activity of the modified hevein-like peptide mAc-AMP2 against susceptible and resistant collection strains of *C. albicans* ATCC 18804 and ATCC 10231, respectively, resistant clinical isolates of *C. albicans* 9.1 or 8.2, and resistant clinical isolates of *C. tropicalis*, *C. krusei,* and *C. glabrata* (Supplementary Materials, Table S1).

**Figure 1:**
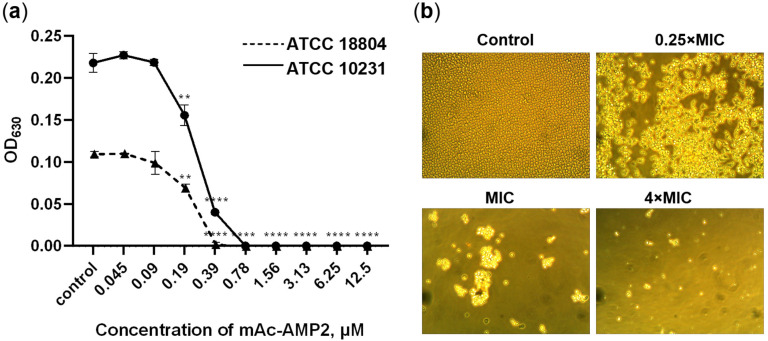
Antifungal activity of mAc-AMP2. (**a**) Effects of mAc-AMP2 at various concentrations on the growth of susceptible and resistant strains of *C. albicans* ATCC 18804 and ATCC 10231, respectively. (**b**) Microscopic analysis of the effects of mAc-AMP2 at various concentrations on *C. albicans* ATCC 18804 cells (400× magnification). Error bars represent a standard deviation (±SD) between technical replications. The untreated controls and samples treated by mAc-AMP2 were compared by an unpaired two-sample *t*-test; significance levels are ** *p* < 0.01, **** *p* < 0.0001.

We showed that mAc-AMP2 was equally effective against all test microorganisms in Sabouraud broth. The MIC values for all fungal strains were 0.39 μM or 0.78 µM (Table 1, Figure 1a). In addition, mAc-AMP2 was shown to be fungicidal against the susceptible strain of *C. albicans* ATCC 18804 and fungistatic against resistant strains of *C. albicans*, *C. tropicalis*, *C. krusei*, and *C. glabrata*. The MFC was 0.78 μM for the *C. albicans* ATCC 18804 and 6.25 μM or above for other tested fungi. Microscopic analysis revealed the absence of *C. albicans* ATCC 18804 growth or lysis of fungal cells at the peptide concentrations of MIC and 4 × MIC, respectively (Figure 1b). Previously, it was shown that Ac-AMP2 inhibited the growth of *C. albicans* at the MIC of 0.87 μM [6].

Various studies have demonstrated that the effectiveness of many cationic AMPs is considerably decreased when exposed to salt concentrations comparable to physiological conditions and in the presence of serum [20,21]. However, evidence suggests that AMPs may indeed be effective in vivo. For instance, histatin-5, a cationic histidine-rich salivary peptide of human and higher primates has demonstrated the efficacy in animal models of oral and vulvovaginal candidiasis [22,23]. Defensin from radish seeds, RsAFP2, has exhibited efficacy in prophylactic murine models of systemic candidiasis [24].

We showed that the presence of 150 mM NaCl inhibited the antifungal activity of mAc-AMP2 but the MIC and the MFC values did not exceed 6.25 µM for *C. albicans* ATCC 18804. The activity of mAc-AMP2 less drastically decreased in the presence of 1.25 mM MgCl_2_ or 1.25 mM CaCl_2_, and the MICs of 1.56 µM or 3.13 µM, respectively, were observed. Surprisingly, the presence of 10% FBS had virtually no effect on the activity of the peptide and MIC and MFC values of 0.78 µM were recorded. pH changes in the range of 5–7 did not affect the activity of this cationic peptide. At the same time, activity of mAc-AMP2 decreased significantly in the nutrient-rich medium RPMI-1640 (Table 2). It is worth noting that endogenic AMPs, human cathelicidin LL-37 and β-defensin HBD2, did not exhibit antifungal activity in this medium at a concentration of 50 μM (MICs 12.5 and 6.25 μM in Sabouraud broth, respectively) (Supplementary Materials, Table S4).

Thus, we demonstrated that mAc-AMP2 at nanomolar concentrations was effective against all tested susceptible and resistant to azoles and other conventional antimycotics strains of *Candida albicans* and non-albicans *Candida* species. The modified hevein-like peptide exhibited fungicidal or fungistatic effects depending on the fungal strain and caused cell lysis at concentrations exceeding its MIC. The anticandidal activity of mAc-AMP2 decreased in the presence of different salts and serums but still remained quite high.

### 3.2. mAc-AMP2 Acts Additively or Independently with Convectional Antimycotics, Ebselen, and Other AMPs

Combination therapy, in which antifungal compounds enhance each other’s effects, is a promising strategy to lower effective doses and, consequently, reduce the side effects of the drugs used. It has been previously shown that Ac-AMP2 action against phytopathogenic fungi was not enhanced when combined with agglutinin, chitinase, glucanase, thionin, or nikkomycin Z [9]. Ac-AMP2 exhibited a synergistic effect against *Penicillium chrysogenum* with thaumatin, which suppresses the growth of hyphae and the formation of fungal spores [6].

Here, we studied the combined action of mAc-AMP2 against *C. albicans* strains with conventional antimycotics (the echinocandin caspofungin and the azole derivative voriconazole) which are administer to treat various types of candidiasis and other substances characterized by pronounced activities and various mechanisms of antifungal action, namely the synthetic organoselenium compound ebselen, endogenic AMPs (human cathelicidin LL-37 and β-defensin HBD2), and tobacco defensin NaD1 [14].

We showed that mAc-AMP2 acted independently in combinations with caspofungin or voriconazole, targeting the fungal cell wall or membrane, respectively (Table 3). Additively action was observed for the combination of mAc-AMP2 with ebselen, which inhibits the fungal plasma membrane H^+^-ATPase proton pump [25], and human cathelicidin LL-37, human β-defensin HBD2 and tobacco defensin NaD1 targeting fungal cell membrane. The lowest FICI of 0.75 was registered for the combinations of mAc-AMP2 with NaD1 or ebselen (Table 3).

Thus, we investigated combinations of mAc-AMP2 with antifungal agents having different mechanisms of action, including conventional antimycotics, other AMPs and organoselenium compound. However, only additive effects against *C. albicans* were registered for the combination of the peptide with ebselen, tobacco defensin NaD1, LL-37, or HBD2. To further characterize the antifungal potential of mAc-AMP2 against *C. albicans* its anti-adherent properties and anti-biofilm activity were investigated.

### 3.3. mAc-AMP2 Inhibits the Adhesion of C. albicans Cells to Epithelial Monolayer and Plastic Surface

**Figure 2:**
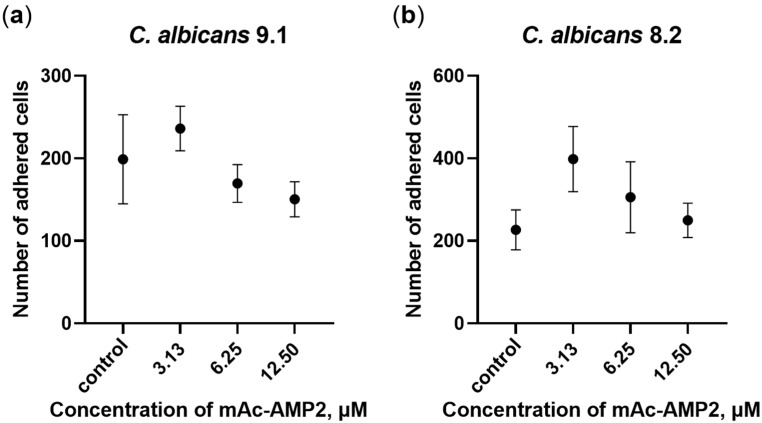
Effects of mAc-AMP2 on *C. albicans* 9.1 (**a**) and 8.2 (**b**) adhesion to the Caco-2 cell monolayer. Error bars represent a standard deviation (±SD) between technical replications.

It is known that adhesion facilitates attachment and colonization of fungal cells on the epithelium of the oral cavity, vagina, and intestines, which can lead to the formation of fungal biofilms and subsequent pathogen invasion. In this study, we examined the influence of mAc-AMP2 on the ability of resistant strains of *C. albicans* ATCC 10231, 9.1 and 8.2 to adhere to the Caco-2 cell monolayer as an in vitro model of human intestinal barrier. Nutrient-rich RPMI-1640 was used in these experiments in which mAc-AMP2 does not exhibit pronounced antifungal activity. We showed that mAc-AMP2 inhibited adhesion of only *C. albicans* 9.1 at the concentrations of 6.25 and 12.5 μM, but this effect was not significant (Figure 2a; Supplementary Materials, Table S5). On the contrary, an increase in the adhesion capacity of clinical isolate 8.2 cells in the presence of the peptide was detected, possibly due to an increase in fungal virulence (Figure 2b). We previously demonstrated that under the same conditions, the tobacco defensin NaD1 effectively inhibited the adhesion of both of these strains at the concentrations of 12.5 μM (2 × MIC) and 25 μM (4 × MIC) [14].

**Figure 3:**
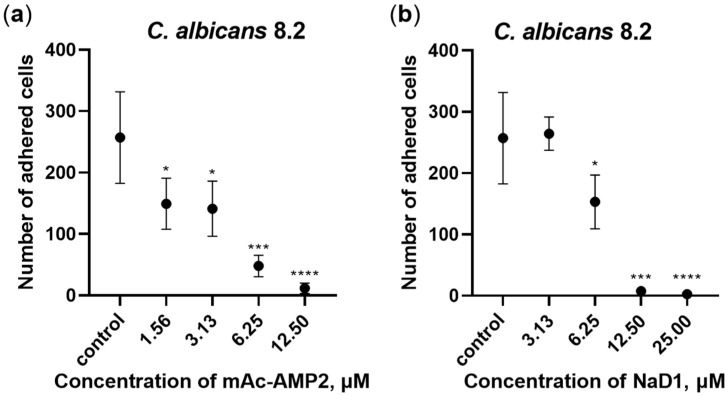
Inhibition of *C. albicans* 8.2 adhesion to the plastic surface by mAc-AMP2 (**a**) and NaD1 (**b**). Error bars represent a standard deviation (±SD) between technical replications. Significance levels are * *p* ≤ 0.05, *** *p* < 0.001 and **** *p* < 0.0001. Untreated control and samples treated by mAc-AMP2 or NaD1 were compared by unpaired two-sample *t*-test.

Adhesion of *C. albicans* cells to various medical devices, including catheters, and the subsequent biofilm formation pose a risk due to the potential development of systemic fungal infections. An ability of mAc-AMP2 to inhibit adhesion of *C. albicans* 8.2 cells to the plastic surface in Sabouraud broth was studied by using 96-well microplates. The tobacco defensin NaD1 as well as caspofungin were used in these experiments. We demonstrated that mAc-AMP2 (MIC 0.78 μM) inhibited adhesion of the clinical isolate 8.2 by 42, 45, 81, and 96% at concentrations of 1.56, 3.13, 6.25, and 12.5 μM, respectively (Figure 3a; Supplementary Materials, Table S6, Figure S7). In comparison, caspofungin inhibited adhesion of fungal cells by approximately 40% at the concentration of 5.12 μg/mL which was more than 100 times higher than its MIC (0.04 μg/mL) (Supplementary Materials, Figure S7). At the same time, NaD1 (MIC 6.25 μM) caused almost 100% inhibition of fungal adhesion also at the concentration of 12.5 μM, which was probably due to its rapid fungicidal action (Figure 3b; Supplementary Materials, Table S6, Figure S7).

Thus, we demonstrated that mAc-AMP2 successfully inhibited the adhesion of *C. albicans* to the plastic surfaces but was considerably less effective at preventing fungal cell attachment to the Caco-2 cell monolayer, mimicking the human intestinal epithelium. However, this difference may be due to the use of various culture media in which both tests were performed.

### 3.4. mAc-AMP2 Exhibits Antibiofilm Activity

**Figure 4:**
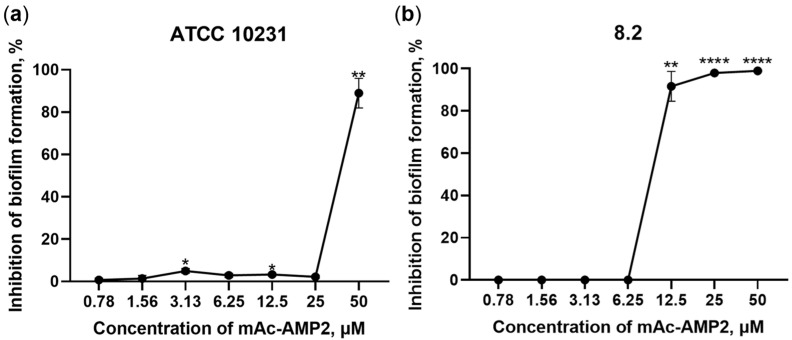
Inhibition of fungal biofilm formation by mAc-AMP2: (**a**) *C. albicans* ATCC 10231; (**b**) *C. albicans* 8.2. Error bars represent a standard deviation (±SD) between technical replications. The untreated controls and samples treated by mAc-AMP2 were compared by unpaired two-sample *t*-test; significance levels are * *p* ≤ 0.05, ** *p* < 0.01, **** *p* < 0.0001.

Next step, we studied the ability of mAc-AMP2 to prevent biofilm formation by *C. albicans*. Resistant strains of *C. albicans* ATCC 10231, 8.2 and 9.1 were used in these experiments. It was shown that mAc-AMP2 inhibited the formation of biofilms by all three resistant fungal strains, but *C. albicans* 8.2 was the most sensitive to the presence of the peptide. In the presence of mAc-AMP2, biofilms either failed to form or developed loosely and got rinsed away during PBS washing of the wells. Nevertheless, some individual fungal cells remained attached in certain wells after rinsing. The BIC values, corresponding to 100% inhibition of biofilm formation, were 12.5 or 50 μM for strain 8.2 or ATCC 10231 and 9.1, respectively (Table 1, Figure 4). The ability of mAc-AMP2 to eradicate already formed biofilms of resistant strains of *C. albicans* ATCC 10231, 8.2 or 9.1 was also tested, but inhibitory effects were hardly observed at the peptide concentrations used.

Thus, we showed that mAc-AMP2 inhibited biofilm formation by resistant strains of *C. albicans*. To the best of our knowledge, this study is the first to demonstrate the anti-adherent and antibiofilm activity of plant hevein-like peptides. To gain insight into the mechanism of action of the modified hevein-like peptide, we investigated the effect of mAc-AMP2 on the chitin content in a *C. albicans* cell wall and its ability to increase fungal cell membrane permeability.

### 3.5. mAc-AMP2 Affects Chitin Content in C. albicans Cell Wall

Previously, the chitin-binding capacity of Ac-AMP2 (identical peptide, designated as Ay-AMP, was isolated from *Amaranthus hypochondriacus* seeds) was demonstrated by using microcolumns packed with chitin [6,9]. Chitinase activity of this peptide was determined by using 4-nitrophenyl-N,N′-diacetyl-β-D-chitobioside as substrate [6]. It was shown that the Ac-AMP2 F18W analogue obtained by solid-phase synthesis, exhibited a higher affinity for chitin than the natural peptide in experiments with chitin bead slurry [13]. Furthermore, an increased chitin-binding capacity of Ac-AMP2 F18W analogue was confirmed by NMR spectroscopy, DYANA molecular modelling, and AMBER-based molecular dynamics of peptide complex with chitin-derived trisaccharide [11]. It is also worth noting that Trp18 is present in the structures of some hevein-like peptides, such as hevein from *Hevea brasiliensis* and Pn-AMP from *Pharbitis nil* [5]. Given that mAc-AMP2 contains only the M13A additional substitution, we hypothesized that the peptide might also possess notable chitin-binding activity.

**Figure 5:**
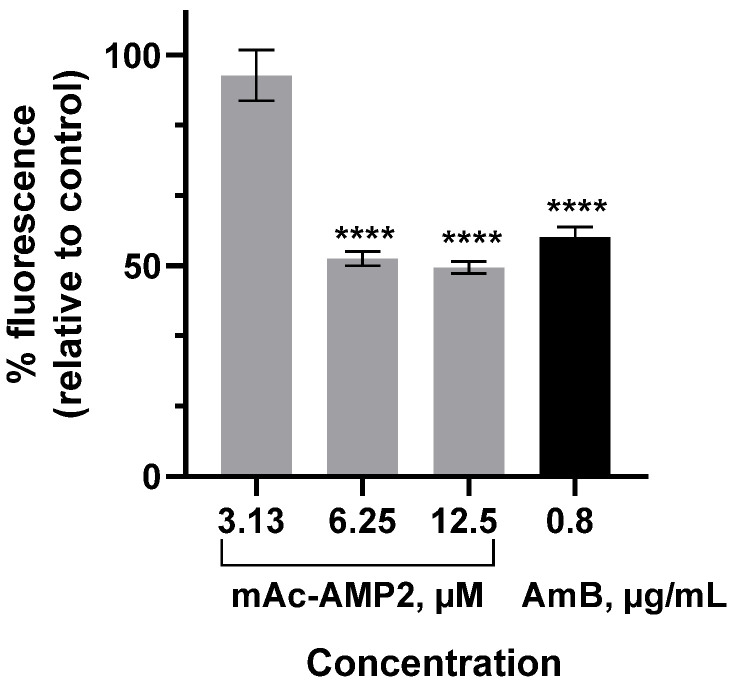
Calcofluor White binding assay. % Fluorescence intensities of *C. albicans* ATCC 18804 cells treated by AmB or mAc-AMP2 at different concentrations relative to untreated fungal cells. Error bars represent a standard deviation (±SD) between technical replications. The untreated and treated cells were compared by unpaired two-sample *t*-test; significance level is **** *p* < 0.0001.

Here, we investigated the ability of mAc-AMP2 at different concentrations to affect chitin content in the *C. albicans* cell wall using fluorescence dye Calcofluor White that binds to chitin. For that *C. albicans* ATCC 18804 cells were incubated in the absence or presence of the peptide at different concentrations for 4 h and fluorescence intensities of Calcofluor White-stained cells were measured by fluorescence spectroscopy. It was shown that the relative fluorescence intensities of stained fungal cells treated by mAc-AMP2 at concentrations of 3.13, 6.25, and 12.5 μM were 93, 50.2, or 46.6%, respectively, compared to the untreated control, indicating that peptide affects cell wall chitin (Figure 5). Fluorescence intensity of *C. albicans* cells treated with amphotericin B (AmB) at MIC was also reduced (Figure 5). Perhaps this effect stemmed from the previously described ability of AmB to inhibit chitin synthase activity [26]. It is important to note that obtained data are preliminary, and further experiments are needed to elucidate mAc-AMP2 effects on the fungal cell wall.

### 3.6. mAc-AMP2 Increases Fungal Cell Membrane Permeability at High Concentrations

**Figure 6:**
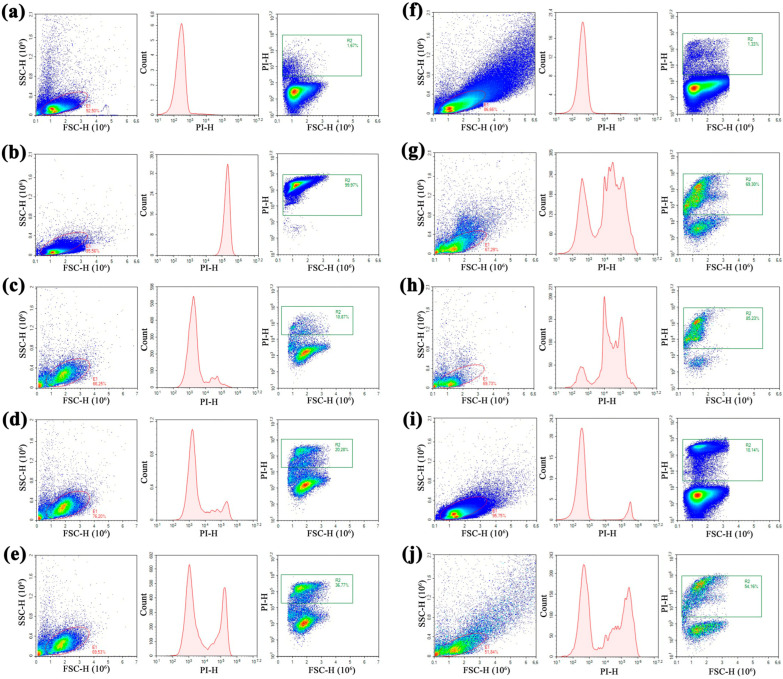
Flow cytometry analysis of the influence of mAc-AMP2, tobacco defensin NaD1, and their combination on the viability of *C. albicans* ATCC 18804 cells, measured by PI uptake: (**a**,**b**)—live and heat-killed cells after incubation for 20 h taken as negative and positive controls, respectively; (**c**–**e**)—cells after incubation for 2 h with mAc-AMP2 at 0.25 × MIC, MIC and 4 × MIC, respectively; (**f**–**h**)—cells after incubation for 20 h with mAc-AMP2 at 0.25 × MIC, MIC and 4 × MIC, respectively; (**i**)—cells after incubation for 20 h with NaD1 at 0.25×MIC; (**j**)—cells after incubation for 20 h with combination of NaD1 and mAc-AMP2 at 0.25 × MIC (FICI 0.5). Events on PI vs. count and FSC vs. PI plots are gated from the FSC vs. SSC diagram.

It has been shown that the mechanism of action of hevein-like peptides including Ac-AMP2 is associated with their chitin-binding and chitinase activities, which lead to disruption of fungal cell wall and fungal death [5]. However, an ability of these peptides to also inhibit the growth of fungi whose cell wall lacks chitin (for example, oomycete pathogen *Phytophthora capsica* [27]) and gram-positive bacteria (for example, *Bacillus megaterium* [9]) possibly suggests the presence of their other targets. For example, it has been demonstrated that wheat hevein-like peptides inhibited the activity of metalloproteinase fungalysin which is an important virulence factor of *Fusarium verticillioides* [28]. Here, we used flow cytometry and intercalating dye propidium iodide (PI), capable of penetrating dead or dying cells with damaged membranes, to study an ability of mAc-AMP2 to affect *C. albicans* ATCC 18804 cell membrane permeability [29]. This rapid, high-throughput method allows for quantification of the effect of AMPs on the cell membrane integrity within a large population of cells [30]. For that, fungal cells were stained by PI after mAc-AMP2 treatment for 2 h. The increase in PI fluorescence was registered as a shift in the peak along the x-axis in the case of heat-killed *C. albicans* cells (Figure 6a,b; Supplementary Materials, Figure S8).

We showed that mAc-AMP2 at the concentrations of 0.25 × MIC and MIC almost did not cause damage to the fungal cell membrane (Figure 6c,d). Surprisingly, for mAc-AMP2 at the concentration of 4 × MIC (1.56 μM) the stained PI cells accounted for 37% which indicated the peptide ability to disrupt integrity of fungal cell membrane at high concentrations (Figure 6e). Under the same conditions, we have previously observed no effects for caspofungin, the main target of which is the fungal cell wall as well as for amphotericin B that binds to ergosterol in the fungal cell membrane [14,31]. To test whether this effect would become more pronounced with increasing exposure time, we incubated *C. albicans* ATCC 18804 cells in the presence of mAc-AMP2 at various concentrations for 20 h. Our assumption was confirmed, and although the peptide did not affect the penetration of the dye at 0.25 × MIC, the number of PI-stained fungal cells was almost 70 and 85% at the peptide concentrations of MIC and 4 × MIC, respectively (Figure 6f–h). Previously, we have shown that in the case of caspofungin an increasing exposure time did not lead to an obvious shift in the peak along the x-axis [14]. This difference in effects, in our opinion, also indicates that mAc-AMP2 has membranotropic activity at its high concentrations. mAc-AMP2 has quite a lot of basic residues in its amino acid sequence (pI 8.92), which is atypical for all hevein-like peptides and indicates a possible affinity of the peptide for the negatively charged components of the fungal cell membrane. It is worth noting that in the case of incubation of *C. albicans* cells with mAc-AMP2 at the peptide concentration of 0.25 × MIC for 20 h, the FSC vs. SSC diagram revealed morphological heterogeneity of fungal cells, which could be the result of their lysis and/or aggregation (Figure 6f). This effect was clearly not observed at higher concentrations of the peptide inhibiting intensive growth of the cell culture (Figure 6g,h).

We also examined effects of the combination of mAc-AMP2 and the membrane-active tobacco defensin NaD1 for which additive effects on *C. albicans* ATCC 18804 cells were registered (Table 3). Both peptides were used at the concentration of 0.25 × MIC in which the percentages of PI-stained cells in the corresponding gate after incubation for 20 h were negligible (1.3% and 10.1% for mAc-AMP2 and NaD1, respectively). However, the percentage of PI-stained cells increased up to 55% in the presence of the combination of mAc-AMP2 and NaD1 (Figure 6i,j). This effect may be due to the disruption of the integrity of the fungal cell wall under the influence of mAc-AMP2 and the facilitation of NaD1 penetration into the cell membrane, or as a consequence of the combined action of two peptides on the fungal membrane.

Thus, we demonstrated that mAc-AMP2 at high concentrations increased *C. albicans* cell membrane permeability. It is possible that at low concentrations the antifungal effects of mAc-AMP2 are mainly due to its chitin-binding capacity, whereas at high concentrations the peptide is capable of exerting membranotropic effects. However, further experiments are needed to confirm this assumption. To evaluate the cytotoxic properties of the peptide, we further investigated its hemolytic activity, as well as the cytotoxic effects on human peripheral blood mononuclear cells (PBMCs), a heterogeneous population of immune cells, and the Caco-2 cell monolayer, which functionally resembles the small intestinal epithelium.

### 3.7. mAc-AMP2 Does Not Exhibit Hemolytic Activity and Cytotoxic Effects Against Caco-2 Cell Monolayer and PBMCs

**Figure 7:**
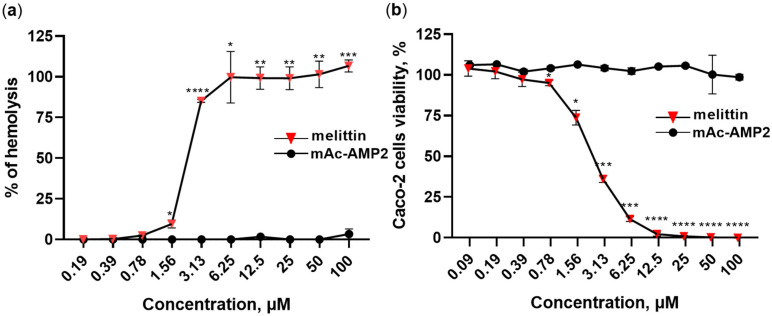
Hemolytic activity (**a**) and cytotoxic effects (**b**) of mAc-AMP2 towards the Caco-2 cell monolayer. Membrane-active melittin from the venom of honeybees was used for comparison. Error bars represent a standard deviation (±SD) between technical replications. Untreated control and samples treated by mAc-AMP2 or melittin were compared by unpaired two-sample *t*-test; significance levels are * *p* ≤ 0.05, ** *p* < 0.01, *** *p* < 0.001 and **** *p* < 0.0001.

Hemolytic activity of mAc-AMP2 was determined using freshly isolated human erythrocytes. mAc-AMP2 did not cause lysis of erythrocytes after incubation for 2 h even at the concentration of 100 µM which is 256 times higher than its average MIC. For comparison, the membrane-active peptide melittin from the honeybee venom caused lysis of about 100% of erythrocytes at the concentration of 6.25 µM (Figure 7a). We showed that mAc-AMP2 also did not exhibit cytotoxic activity. A cell viability of approximately 100% was observed even at the peptide concentration of 100 µM in the case of the Caco-2 cell monolayer. At the same time, melittin induced approximately 100% cell death at the concentration of 12.5 µM (Figure 7b). The absence of cytotoxic effects was also shown for PBMCs at a mAc-AMP2 concentration of 25 µM (Supplementary Materials, Figure S9).

It is well known that many AMPs exhibit a high toxicity, which limits their potential as new antifungal agents. At the same time, despite high anticandidal activity, mAc-AMP2 does not exhibit cytotoxic effects even at concentrations many times higher than its MICs, which is probably due its preferential action on fungal-specific targets. The obtained results are consistent with the previously described data for Ac-AMP2 and the peptide with the same sequence Ay-AMP from *Amaranthus hypochondriacus*. It was shown that these peptides were not toxic for T lymphocytes, human umbilical endothelial cells, or human skin-muscle fibroblasts [6,9].

### 3.8. Stability of mAc-AMP2

It has been previously shown that the activity of Ac-AMP2 against phytopathogenic fungi is preserved upon boiling for 10 min, by exposure to extreme pH values of 2.0 or 11.0, after treatment with such proteases as pepsin, papain, chymotrypsin, proteinase K, or pronase E, but not trypsin, which increased the IC_50_ values by about 10-fold [6,9]. At the same time, reduction in disulfide bonds by dithiothreitol (DTT) resulted in loss of antifungal activity [6,9]. Here, we investigated the effects of heating and the reducing agent DTT on peptide anticandidal activity, as well as the stability of mAc-AMP2 to serum enzymes.

The activity of mAc-AMP2 against the susceptible strain of *C. albicans* ATCC 18804 reduced after heating at 99 °C for 15 min, and its MIC and MFC values increased by two or four times, respectively. The presence of reduced glutathione (GSH), a component of RPMI-1640 medium, at low concentration of 3.25 μM increased the MIC and MFC of the peptide by half. At the same time, treatment of the peptide with excess DTT resulted in loss of activity, and no effects on *C. albicans* growth were observed even at its concentration of 12.5 μM. At the same time, the antifungal activity of tobacco defensin NaD1, whose structure is also stabilized by disulfide bonds, was less sensitive to heating and DTT treatment. Interestingly, the MIC value of NaD1 did not change under these conditions, but the peptide action became less fungicidal (Table 4).

The tertiary structure of Ac-AMP2 is stabilized by three disulfide bonds and consists of a N-terminal loop, a centrally located twisted anti-parallel β-sheet acting as the core of the peptide, and a C-terminal helical turn [10]. The NaD1 structure is characterized by the cysteine-stabilized αβ (CSαβ) motif consisting of a triple-stranded antiparallel β-sheet and a single α-helix, reinforced by four disulfide bonds. It has been shown that NaD1-C3S-C47S variant lacking the fourth disulfide bond exhibited similar antifungal activity as that of the unmodified tobacco defensin [32] while reduction and alkylation of NaD1 inactivated the peptide [33]. Probably, NaD1 has a compact structure that is stabilized not only by disulfide bonds, and their reduction does not lead to peptide denaturation, unlike in the case of mAc-AMP2.

**Figure 8:**
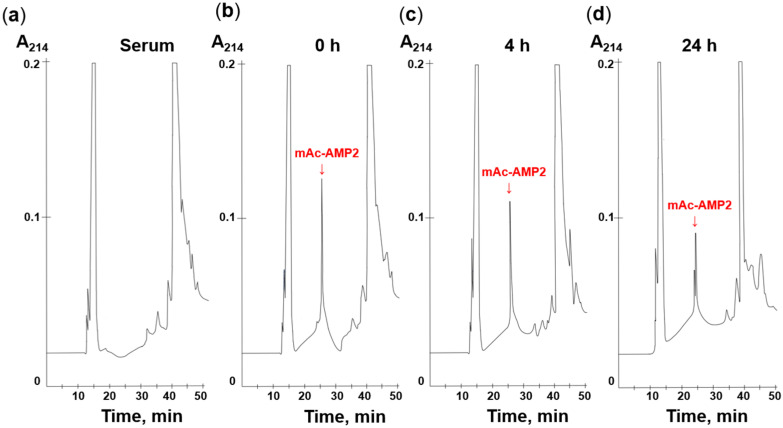
RP-HPLC analysis of mAc-AMP2 degradation by serum (**a**) for 0 (**b**), 4 (**c**), and 24 h (**d**).

To examine stability of mAc-AMP2 in serum, the peptide was incubated for 0, 4, or 24 h in the presence of 25% freshly isolated human serum. After that, the serum components were precipitated with TFA in the presence of urea, and RP-HPLC analysis of resulting supernatants was performed. It was shown that incubation with serum caused a reduction in mAc-AMP2 concentration over time. However, even after 24 h, the peptide’s characteristic peak was still detectable in the chromatogram (Figure 8, Supplementary Materials, Figure S6). Thus, we demonstrated that mAc-AMP2 is quite stable in the presence of serum enzymes and maintains high antifungal activity in the presence of serum. However, its potential efficacy with systemic administration remains questionable due to the sensitivity of antifungal activity to the presence of salts.

### 3.9. Decrease in Sensitivity of C. albicans to mAc-AMP2

**Figure 9:**
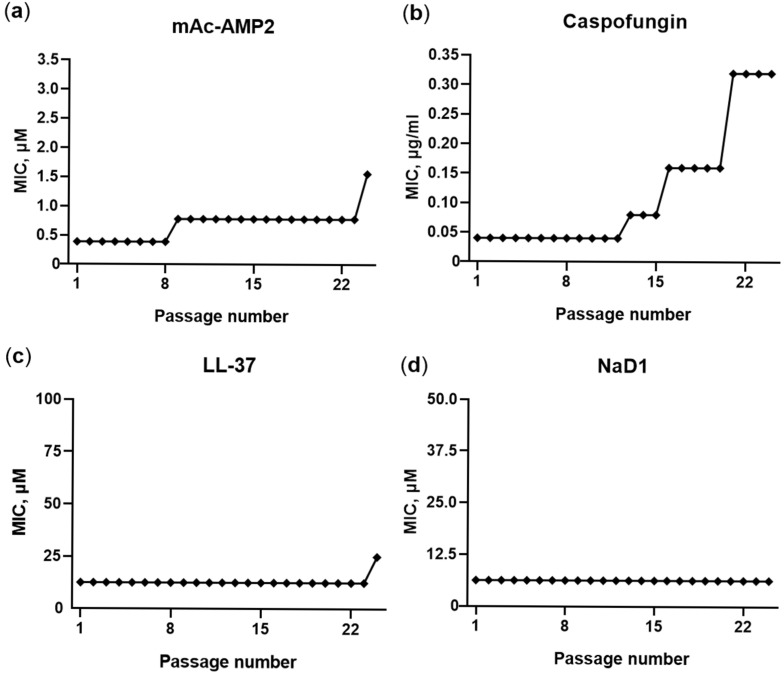
Serial passage of *C. albicans* ATCC 18804 in the presence of mAC-AMP2 (**a**), caspofungin (**b**), LL-37 (**c**), and NaD1 (**d**) to assess the potential for the development of resistance.

As shown, the decrease in sensitivity of fungi of the *Candida* genus upon prolonged and repeated exposure to an antifungal agent contributes to treatment failures and infection recurrence. Therefore, the ability of fungal cells to develop resistance to mAc-AMP2 was investigated. The endogenic human antimicrobial peptide LL-37 and the tobacco defensin NaD1, as well as the conventional antimycotic caspofungin were tested in comparison. The susceptible strain of *C. albicans* ATCC 18804 and the resistance one ATCC 10231 were used in these experiments. Fungal cells were cultured by sequential passaging of *C. albicans* strains in the presence of subinhibitory concentrations of antimicrobial agents. The results obtained were similar for both strains of *C. albicans*. During the experiment, the MIC of mAc-AMP2 against *C. albicans* ATCC 18804 consistently increased four-fold (from 0.39 to 1.56 μM) after the 8th and 24th passages (Figure 9a). At the same time, the resistance to caspofungin was registered later, but resulted in an eight-fold MIC increase after 21 passages (Figure 9b). In contrast, resistance to the membrane-active LL-37 developed slowly and only two-fold MIC increase was registered after the 24th passage, while no change in the sensitivity of *C. albicans* to the tobacco defensin NaD1 was observed (Figure 9c,d). Thus, the sensitivity of *C. albicans* to mAc-AMP2 decreased over time, but the dynamics of this process were similar to those of caspofungin, which also affects fungal cell wall. At the same time, the fungal sensitivity in actual fact did not change in the presence of LL-37 and NaD1, the main target of which is the fungal cell membrane [14].

*C. albicans* cells of both strains capable of growing at the highest concentration of mAc-AMP2 at the end of experiment were passaged thrice on agar plates in the absence of the peptide. After that, whole cultures and separate clones were re-tested for MIC determination. It was found that the MICs returned to their initial values. It is known that decreased sensitivity to antifungal drugs occurs due to the development of fungal resistance, heteroresistance, or tolerance [34]. Fungal resistance typically develops due to genetic mutations. Heteroresistance is caused by the emergence of a small subpopulation of fungal cells that can grow at drug concentrations above the MIC; the appearance of such persistent cells may be associated with gene amplification or aneuploidy as well as with non-genetic changes including chromatin modifications and metabolic shifts. Intrinsic or acquired tolerance allows fungal cells to survive longer under drug exposure and may be caused by the same changes in fungal cells as heteroresistance. It is worth noting that heteroresistance and tolerance may provide only a temporary decrease in sensitivity, and fungal cells may return to a fully susceptible state after antifungal agent elimination [34,35]. The clinical importance of tolerance remains an open question. It is suggested that tolerance may contribute to both recurrent infections and the ability to develop drug resistance [34]. Since the decrease in fungal sensitivity to mAc-Amp2 was not stable, in our case it was unlikely to be the result of mutations and more probable that heteroresistance or acquired tolerance took place.

## 4. Conclusions

Thus, we showed that the modified hevein-like peptide mAc-AMP2 possessed a pronounced antifungal activity at nanomolar concentrations against susceptible and resistant strains of fungi of the *Candida* genus including *C. albicans*. The peptide is small in size and characterized by a low toxicity and a moderate stability. Anticandidal activity of mAc-AMP2 remains quite high in the presence of various salts and serums. This peptide is capable of preventing the adhesion of *C. albicans* cells to plastic surfaces and inhibits the formation of biofilms by the resistant strains of this fungus. Sensitivity of *C. albicans* to mAc-Amp2 may decrease over time; however, this reduction might not be associated with mutations in the fungal genome and could be restored after the peptide elimination. All this together makes mAc-AMP2 a promising drug candidate for the treatment of fungal infections caused by *C. albicans* and non-albicans *Candida* species. However, further studies are needed to clarify the mechanism of mAc-AMP2 action, including its effects on the fungal cell wall and membrane, and other possible extracellular and intracellular targets. Furthermore, animal models with superficial and systemic candidiasis are required to assess its efficacy in vivo.

## Figures and Tables

**Table 1:** Activity of mAc-AMP2 against collection strains and clinical isolates of fungi of the *Candida* genus (the peptide concentrations are stated in μM).

*Candida* Strains	MIC_50_	MIC	MFC	BIC_50_	BIC
*C. albicans* ATCC 18804	0.19–0.39	0.39	0.78	nd	nd
*C. albicans* ATCC 10231	0.19–0.39	0.78	>6.25	25–50	50
*C. albicans* 8.2	0.39	0.78	>6.25	6.25–12.5	12.5
*C. albicans* 9.1	0.19–0.39	0.39	6.25	6.25–12.5	50
*C. tropicalis* v13a4/2	0.09–0.19	0.39	>12.5	nd	nd
*C. krusei* 225/2	0.09–0.19	0.39	>12.5	nd	nd
*C. glabrata* 252/2	0.09–0.19	0.39	>12.5	nd	nd

nd—not determined; MIC_50_ and MIC—minimal inhibitory concentration providing 50% or 100% inhibition of fungal growth, respectively; MFC—minimum fungicidal concentration; BIC_50_ and BIC—minimal inhibitory concentration providing 50 or 100% inhibition of biofilm formation, respectively.

**Table 2:** The influence of different salts and serum on the activity of mAc-AMP2 against *C. albicans* ATCC 188804 (peptide concentrations are presented in μM; medium—½Sabouraud broth, pH 6.0).

Medium	MIC	MFC
No salts, no FBS	0.39	0.78
150 mM NaCl	6.25	6.25
1.25 mM CaCl_2_	3.13	6.25
1.25 mM MgCl_2_	1.56	3.13
10% FBS	0.78	0.78
RPMI-1640	50	>50
pH 5.0	0.39	0.78
pH 7.0	0.39	0.78

MIC—minimal inhibitory concentration providing 100% inhibition of fungal growth; and MFC—minimum fungicidal concentration.

**Table 3:** Activity of mAc-AMP2 combinations with conventional antimycotics, ebselen, or other AMPs against susceptible and resistant strains of *C. albicans*.

Antifungal Agents	*C. albicans* Strain	FICI	[A]/MIC_A_	[B]/MIC_B_
Conventional antimycotics				
Voriconazole *	ATCC 18804	2	1	1
Caspofungin		2	1	1
Antimicrobial peptides				
Human defensin HBD2	ATCC 18804	1	0.5	0.5
Human cathelicidin LL-37	ATCC 18804	1	0.5	0.5
Tobacco defensin NaD1	ATCC 18804	0.75	0.5	0.25
	9.1	1	0.5	0.5
	8.2	1	0.5	0.5
Organic selenium compounds				
Ebselen	ATCC 18804	1	0.5	0.5
	9.1	1	0.5	0.5
	8.2	0.75	0.5	0.25

FICI—fractional inhibitory concentration index determining the type of interaction between two antifungal agents (synergism at FICI ≤ 0.5, additive action at 0.5 < FIC ≤ 1, independent action at 1 < FIC ≤ 2). [A]/[MIC_A_] and [B]/[MIC_B_]—ratios of the concentrations of mAc-AMP2 or other antifungal agent in combination and their MICs, respectively. * For voriconazole MIC_50_ values were used.

**Table 4:** Activity of mAc-AMP2 and tobacco defensin NaD1 against *C. albicans* ATCC 18804 after reduction in -SS- bonds or heating (peptide concentrations are stated in μM).

Peptides	Without		3.25 μM GSH		DTT		99 °C	
	MIC	MFC	MIC	MFC	MIC	MFC	MIC	MFC
mAc-AMP2	0.39	0.78	0.78	1.56	>12.5	nd	0.78	3.13
NaD1	6.25	12.5	nd	nd	6.25	25	6.25	25

nd—not determined; GSH—reduced glutathione; MIC—minimal inhibitory concentration providing 100% inhibition of fungal growth; MFC—minimum fungicidal concentration.

## Data Availability

The original contributions presented in the study are included in the article/Supplementary Materials; further inquiries can be directed to the corresponding author.

## Supplementary Materials

The following supporting information can be downloaded at: https://www.mdpi.com/article/10.3390/pharmaceutics17111406/s1, References [36,37] are cited in the supplementary materials. Figure S1: Agarose electrophoresis of PCR results for the production of amplicons encoding mAc-AMP2 (A) and the quality of the assembled plasmid construction (B); Figure S2: Schematic representation of the plasmid vectors pET-His8-TrxL-mAc-AMP2; Figure S3: Electrophoretic analysis (15% SDS-PAGE) of the expression, isolation and purification of mAc-AMP2; Figure S4: MALDI-TOF mass-spectra of recombinant mAc-AMP2 (calculated molecular weight of the peptide with three SS bonds is 3162.7 Da) before (A) and after modification with iodoacetamide without (B) and after reduction with dithiothreitol (C); Figure S5: Circular dichroism spectra of mAc-AMP2 (0.3 mM) in an aqueous solution; Figure S6: RP-HPLC analysis of mAc-AMP2 incubation without serum for 0 (a), 4 (b) and 24 h (c); Figure S7: Influence of mAc-AMP2, tobacco defensin NaD1 and caspofungin on the ability of the clinical isolate of *C. albicans* 8.2 to adhere onto plastic surface; Figure S8: Flow cytometry analysis the viability of *C. albicans* ATCC 18804 cells, measured by PI uptake; Figure S9: PBMCs viability in the presence of mAc-AMP2 or membrane-active peptide melittin from the venom of honeybees; Table S1: Characteristics of strains of *Candida* used (according to [S1,S2]); Table S2: List of overlapping primers for mAc-AMP2 production; Table S3: mAc-AMP2 secondary structure estimation (%) predicted from far-UV CD spectrum; Table S4: Antifungal activity of endogenic AMPs, human cathelicidin LL-37 and β-defensin HBD2, towards *C. albicans* ATCC 18804; Table S5: Effects of mAcAMP2 on the adhesion of fungal cells to Caco-2 cell monolayer; Table S6: Effects of mAcAMP2 and NaD1 on the adhesion of *C. albicans* 8.2 cells to the plastic surface.
