# Supplementary material for: Modified Hevein-like Peptide from Amaranthus caudatus as a Promising Agent Against Pathogenic Candida Species
Source: Pharmaceutics. 2025 Oct 30;17(11):1406. doi: 10.3390/pharmaceutics17111406 (PMC12655060; doi:10.3390/pharmaceutics17111406)
Supplement: Supplementary file 1 [file pharmaceutics-17-01406-s001.zip › pharmaceutics-3902406-supplementary.pdf]

Supplementary information for

Modified Hevein-like Peptide from *Amaranthus caudatus* as a Promising Agent Against Pathogenic *Candida* Species

Ekaterina I. Finkina [1,2,*], Anastasia A. Gerasimova [1], Olga V. Shevchenko [1,2], Ivan V. Bogdanov [1], Andrey A. Tagaev [1], Alexander D. Voropaev [3] and Tatiana V. Ovchinnikova [1,2]

[1] M.M. Shemyakin and Yu.A. Ovchinnikov Institute of Bioorganic Chemistry, Russian Academy of Sciences, 117997 Moscow, Russia; ger7373@yandex.ru (A.A.G.); sh.o.v.2001@gmail.com (O.V.S.); contraton@mail.ru (I.V.B.); andtag@mx.ibch.ru (A.A.T.); ovch@ibch.ru (T.V.O.)
[2] Moscow Center for Advanced Studies, 123592 Moscow, Russia
[3] G.N. Gabrichevsky Research Institute for Epidemiology and Microbiology, Admiral Makarov St. 10, 125212 Moscow, Russia; advoropaev@gmail.com
* Correspondence: finkina@mail.ru; Tel.: +7-495-335-0900

Materials and Methods

Assembly of genetic construct

DNA fragment encoding mAc-AMP2 (VGECVRGRCPSGACCSQWGYCGKGPKYCGR) was obtained by de novo synthesis using PCR with overlapping primers (Supplementary materials, Table S2) and Tersus polymerase (Evrogen, Russia). The following thermocycling conditions were used: 95°C – 1 min; then 30 amplification cycles (95°C - 30 s, 55° - 15 s, 72°C - 15 s); then 72°C – 2 min. Alternatively, step-down PCR with a decrease in primer annealing temperature by 5 degrees every five cycles was used. The quality of the synthesized DNA fragments for mAc-AMP2 (146 bp) was assessed by electrophoresis in 1.5% agarose gel and ChemiDoc XRS+ gel documentation system (Supplementary materials, Figure S1A).

Then DNA fragment was inserted into the expression plasmid vector pET-His8-TrxL, which was pre-treated with BamHI (Thermo Fisher Scientific, Inc., Waltham, MA, USA). The expression plasmid pET-His8-TrxL-mAc-AMP2 was constructed by the ligase-free cloning method using Antarctic Phosphatase (New England Biolabs, Ipswich, MA, USA). Then the reaction mixtures were used to transform competent *E. coli* DH10B cells. Right plasmid assemble was verified by electrophoresis in 0.8% agarose gel (Supplementary materials, Figure S1B) and DNA sequencing performed in two directions (Supplementary materials, Figure S2).

Recombinant production and purification of mAc-AMP2

Recombinant peptide was obtained by heterologous expression in *E. coli* cells. For that chemically competent BL21(DE3) cells were transformed with expression plasmid pET-His8-TrxL-mAc-AMP2 by heat shock. BL21(DE3)/pET-His8-mAc-AMP2 cells were grown at 37°C in LB medium with 100 μg/mL of ampicillin and 20 mM glucose up to OD_{600} 0.5-0.8 and then were induced with 0.2 mM isopropyl-β-D-thiogalactopyranoside (IPTG). The cells after growth during 4-6 h at 30°C were harvested by centrifugation and sonicated in the buffer containing 20 mM imidazole. Soluble fraction was separated by centrifugation and then loaded into Ni–NTA column. The recombinant protein was eluted with the buffer containing 0.5 M imidazole. After dialysis the protein of interest was dissolved in 80% TFA and then cleaved by an equal mass of CNBr under standard conditions. The carrier protein was eliminated from the mix by the second Ni–NTA chromatography stage. Final purification of mAc-AMP2 was performed by using the RP-HPLC semi-preparative column Reprosil-pur C18-AQ (Dr. Maisch GmbH). The chromatography was performed in the presence of 0.1% TFA in the gradient of acetonitrile 5-80%.

Table S1. Characteristics of strains of *Candida* used (according to [36,37]).

Fungal strains	Polyenes	Azoles	Echinocandins
C. albicans ATCC 18804	S	S	S
C. albicans ATCC 10231	S	R	R*
C. albicans 8.2	S	R	R*
C. albicans 9.1	S	R	R*
C. krusei 225/2	S	R	S
C. tropicalis v13a4/2	R	R	S
C. glabrata 252/2	R	R	S

S – susceptible, R – resistant, R* – resistant only to anidulafungin.

Table S2. List of overlapping primers for mAc-AMP2 production.

№	primer sequences (5′-3′)
Dir1	CCTCGACGCTAACCTGGCCGGATCTATGGTGGGTGAATGTGTGC
Dir2	TATGGTGGGTGAATGTGTGCGTGGTCGTTGCCCAAGTGGCGCATGTTGCAG
Rev1	ACTTTGGGCCTTTACCACAGTAGCCCCACTGACTGCAACATGCGCCACTTGGG
Rev2	GGTGCTCGAGAGAATTCGCGGATCCTTAACGGCCACAGTACTTTGGGCCTTTACCACAGTA

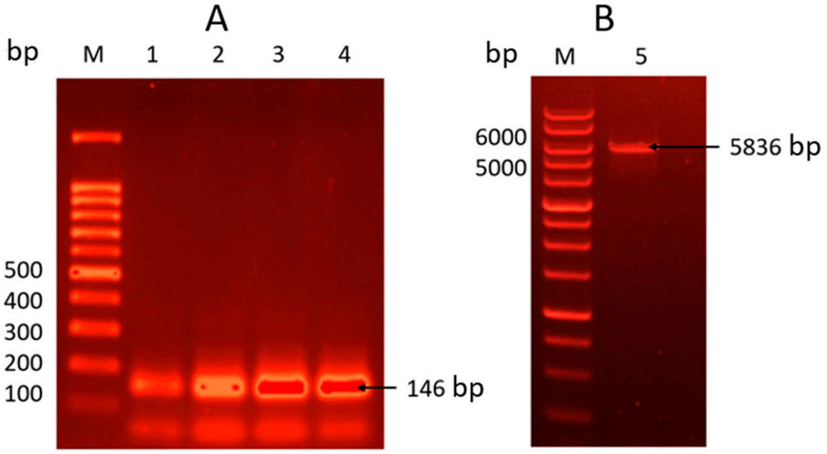

Figure S1. Agarose electrophoresis of PCR results for the production of amplicons encoding mAc-AMP2 (A) and the quality of the assembled plasmid construction (B). M – marker; 1,2,3,4 – PCR products obtained at primer annealing temperatures of 50°C, 55°C, 60°C and using the step-down program, respectively; 5 – plasmid construction pET-His8-TrxL-mAc-AMP2.

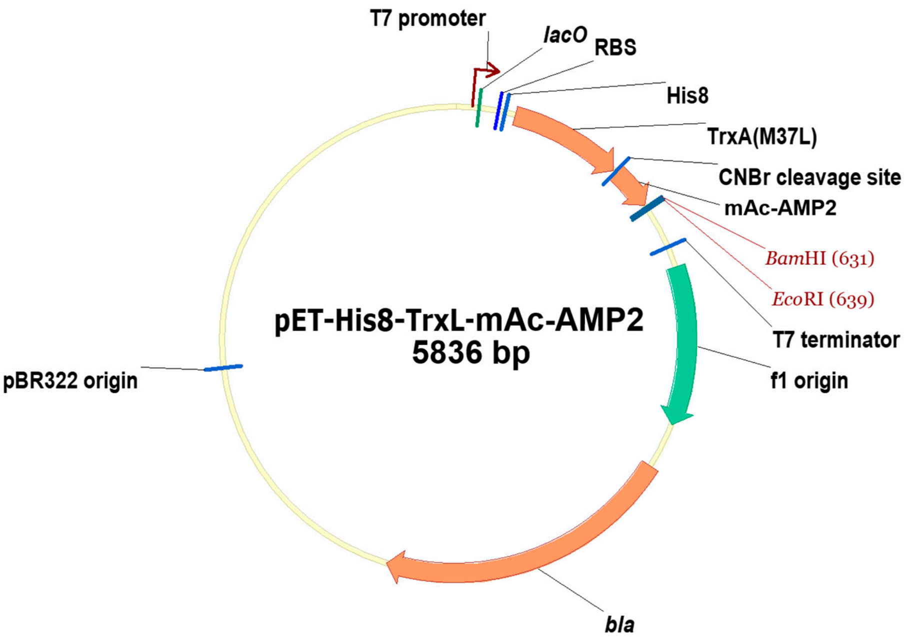

Figure S2. Schematic representation of the plasmid vectors pET-His8-TrxL-mAc-AMP2.

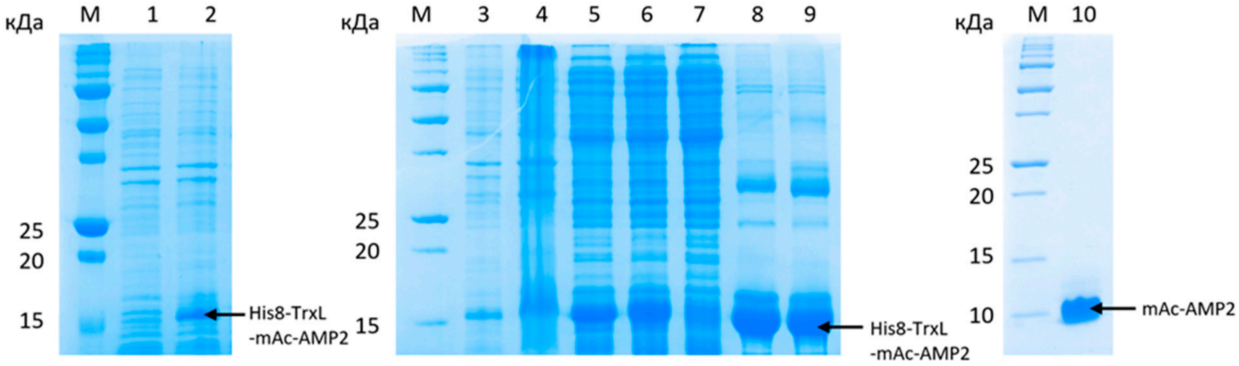

Figure S3. Electrophoretic analysis (15% SDS-PAGE) of the expression, isolation and purification of mAc-AMP2: M – molecular weight protein standards; 1,2 - cellular lysates before and after IPTG induction; 3,4 – insoluble cell fraction without and in the presence of β-mercaptoethanol (BME); 5,6 – soluble cell fraction without and in the presence of BME; 7 – breakthrough after metal chelate chromatography; 8,9 – eluate after metal chelate chromatography without and in the presence of BME; 10 – purified mAc-AMP2.

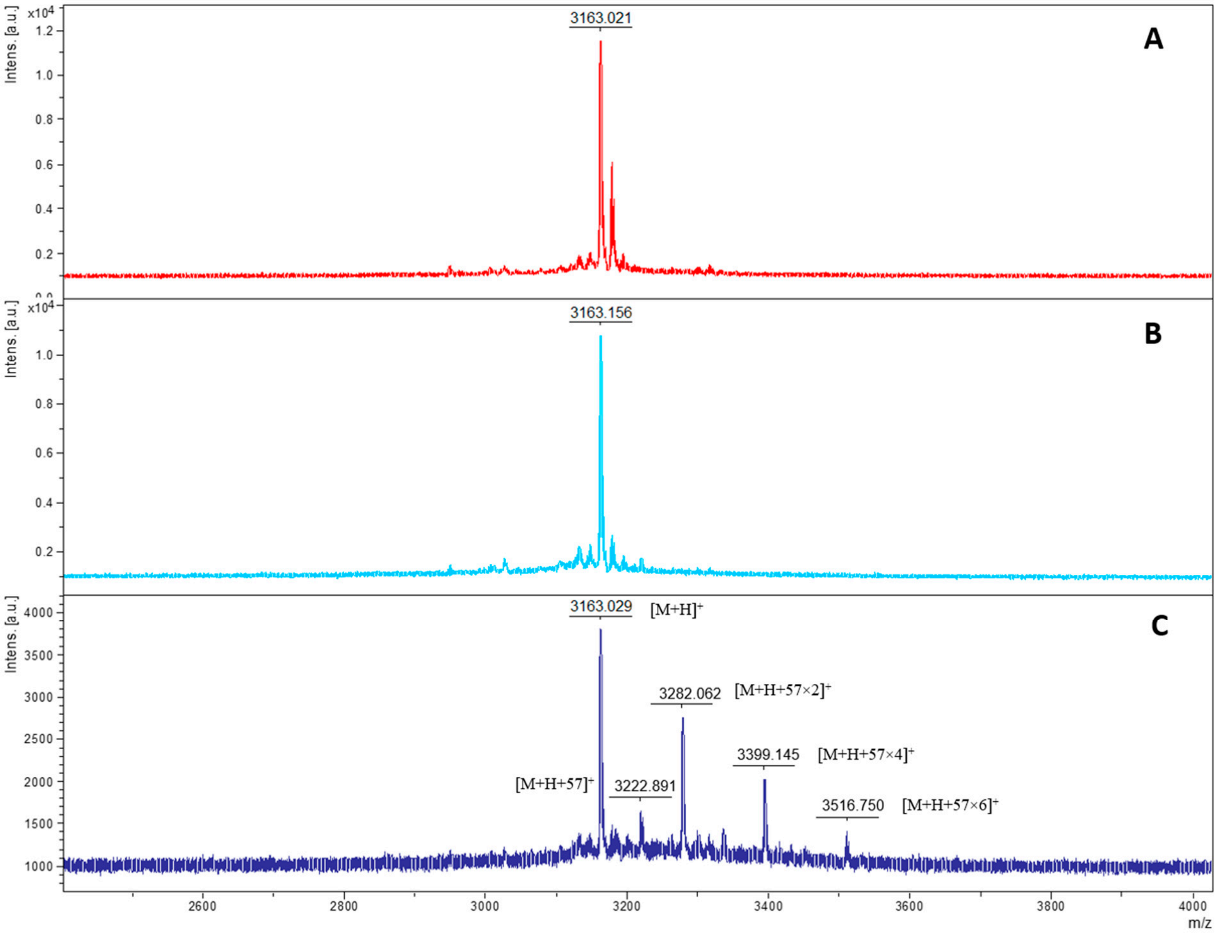

Figure S4. MALDI-TOF mass-spectra of recombinant mAc-AMP2 (calculated molecular weight of the peptide with three SS bonds is 3162.7 Da) before (A) and after modification with iodoacetamide without (B) and after reduction with dithiothreitol (C).

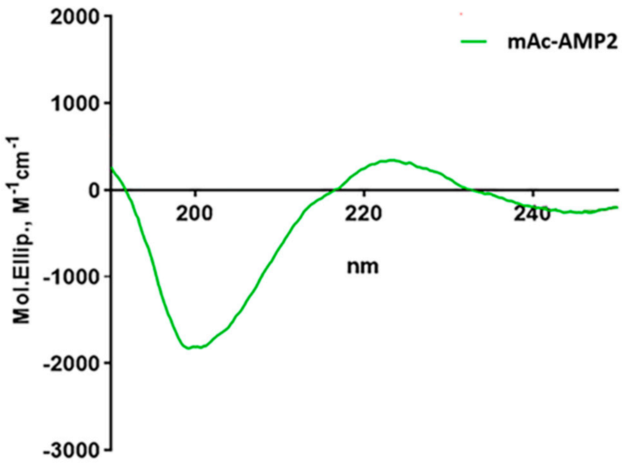

Figure S5. Circular dichroism spectra of mAc-AMP2 (0.3 mM) in an aqueous solution. Measurements were performed at room temperature using a J-810 spectropolarimeter (JASCO Corp., Japan) in a cuvette with an optical path length of 0.01 cm.

Table S3. mAc-AMP2 secondary structure estimation (%) predicted from far-UV CD spectrum.

Peptide	Condition	α-Helix, %	β-Sheet, %	β-Turn, %	Random, %	NRMSD
mAc-AMP2	Aqueous solution	3.4	42.6	21.4	32.5	0.29

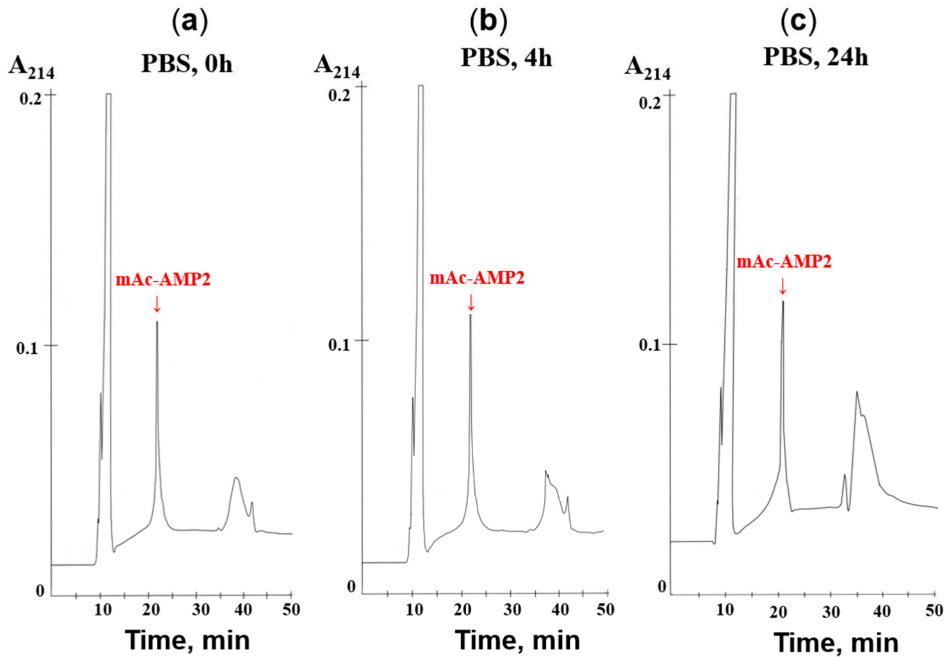

Figure S6. RP-HPLC analysis of mAc-AMP2 incubation without serum for 0 (a), 4 (b) and 24 h (c).

Table S4. Antifungal activity of endogenic AMPs, human cathelicidin LL-37 and β-defensin HBD2, towards *C. albicans* ATCC 18804.

Peptides	MIC, µM	
	½Sabouraud broth	RPMI-1640
LL-37	12.5	>50
HBD2	6.25	>50

Table S5. Effects of mAcAMP2 on the adhesion of fungal cells to Caco-2 cell monolayer.

**Concentration, μM**	***C. albicans* 9.1**	
	**Cell adhesion, %**	**Inhibition of adhesion, %**
**12.5**	30.07	24.39 (ns)
**6.25**	33.93	14.67 (ns)
**3.13**	47.23	-
**control**	39.77	-

ns – non significant.

Table S6. Effects of mAcAMP2 and NaD1 on the adhesion of *C. albicans* 8.2 cells to the plastic surface.

**Concentration, μM**	**mAc-AMP2**		**NaD1**	
	**Cell adhesion, %**	**Inhibition of adhesion, %**	**Cell adhesion, %**	**Inhibition of adhesion, %**
**25**	nd	nd	0.05	99.03 (p=0.0001)
**12.5**	0.23	95.46 (p=0.0002)	0.15	97.12 (p=0.001)
**6.25**	0.96	81.32 (p=0.0006)	0.96	81.32 (p=0.001)
**3.13**	2.83	45.01 (p=0.0127)	5.29	-
**1.56**	2.99	41.89 (p=0.0046)	nd	nd
**control**	5.14	-	5.14	-

nd – not determined. Significance levels are counted by comparison of untreated control and samples treated by mAc-AMP2 or NaD1 by unpaired two-sample t-test.

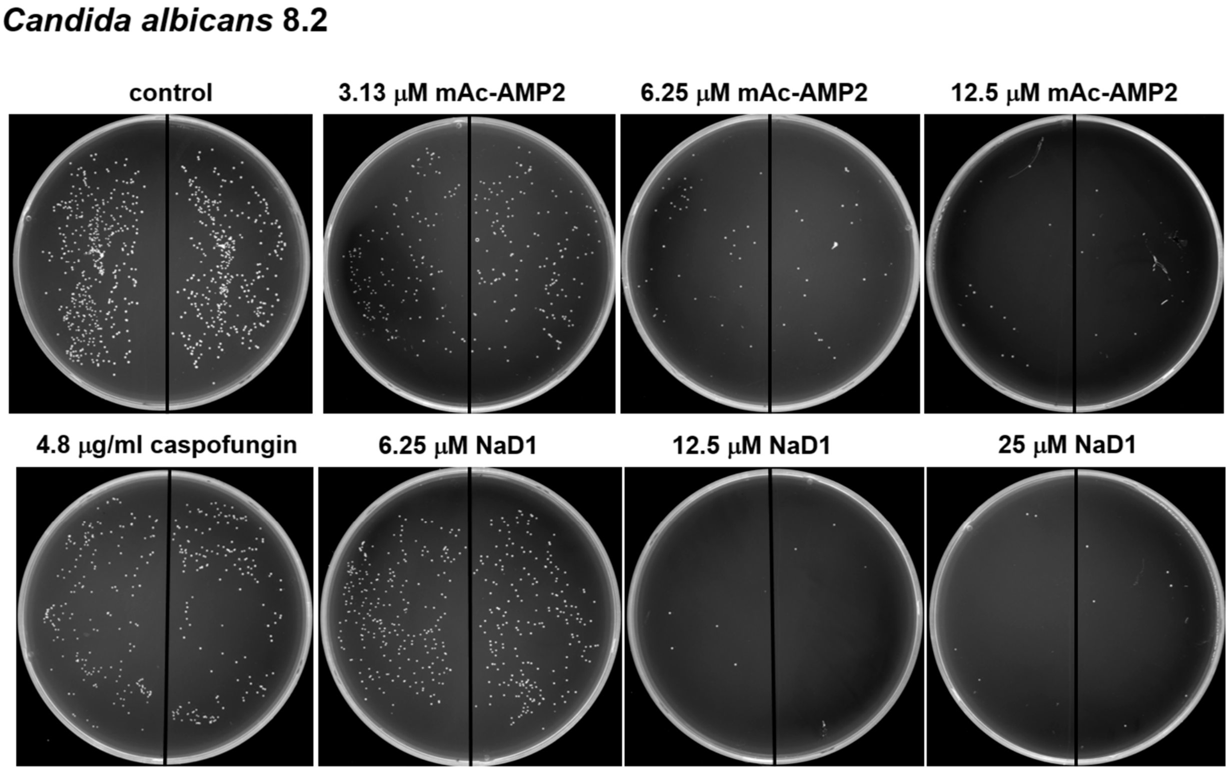

Figure S7. Influence of mAc-AMP2, tobacco defensin NaD1 and caspofungin on the ability of the clinical isolate of *C. albicans* 8.2 to adhere onto plastic surface. The results of plating on Sabouraud agar of detached fungal cells from two technical replicates for each variant are presented.

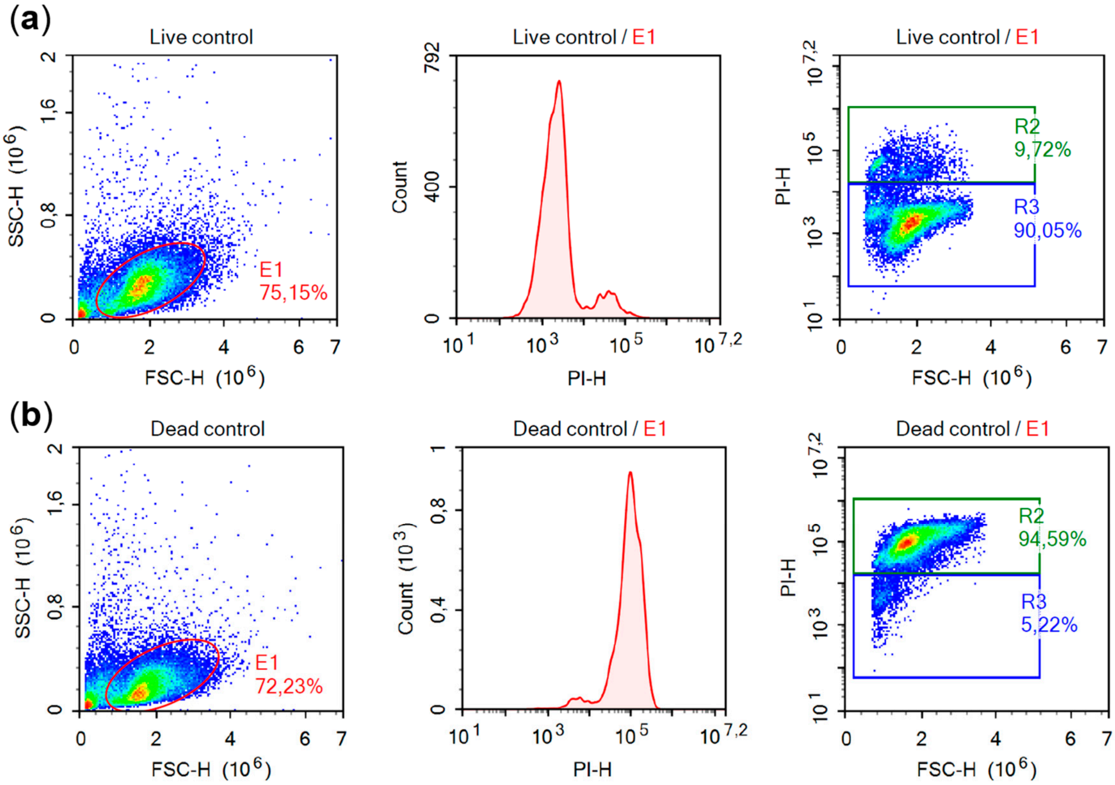

Figure S8. Flow cytometry analysis the viability of *C. albicans* ATCC 18804 cells, measured by PI uptake: (a,b) – live and heat-killed cells after incubation for 2 h taken as negative and positive controls, respectively. Events on PI vs. count and FSC vs. PI plots are gated from the FSC vs. SSC diagram.

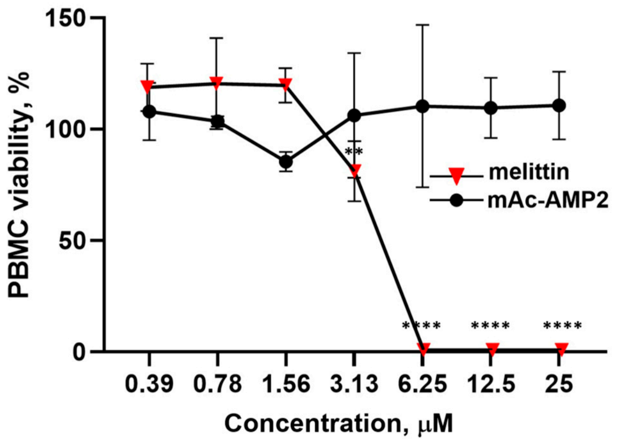

Figure S9. PBMCs viability in the presence of mAc-AMP2 or membrane-active peptide melittin from the venom of honeybees. Error bars represent a standard deviation (±SD) between technical replications. Untreated control and samples treated by mAc-AMP2 or melittin were compared by unpaired two-sample t-test; significance levels are $^{**}p < 0.01$, $^{***}p < 0.001$ and $^{****}p < 0.0001$.
